# Comparative Proteomic Profiling of *Ehrlichia ruminantium* Pathogenic Strain and Its High-Passaged Attenuated Strain Reveals Virulence and Attenuation-Associated Proteins

**DOI:** 10.1371/journal.pone.0145328

**Published:** 2015-12-21

**Authors:** Isabel Marcelino, Miguel Ventosa, Elisabete Pires, Markus Müller, Frédérique Lisacek, Thierry Lefrançois, Nathalie Vachiery, Ana Varela Coelho

**Affiliations:** 1 Instituto de Biologia Experimental e Tecnológica (iBET), Oeiras, Portugal; 2 Instituto de Tecnologia Química e Biológica António Xavier, Universidade Nova de Lisboa (ITQB-UNL), Oeiras, Portugal; 3 CIRAD, UMR CMAEE, F-97170, Petit-Bourg, Guadeloupe, France; 4 INRA, UMR1309 CMAEE, F-34398, Montpellier, France; 5 Proteome Informatics, Swiss Institute of Bioinformatics (SIB), Geneva, Switzerland; 6 CIRAD, UMR CMAEE, F-34398, Montpellier, France; University of Texas Medical Branch, UNITED STATES

## Abstract

The obligate intracellular bacterium *Ehrlichia ruminantium* (ER) causes heartwater, a fatal tick-borne disease in livestock. In the field, ER strains present different levels of virulence, limiting vaccine efficacy, for which the molecular basis remains unknown. Moreover, there are no genetic tools currently available for ER manipulation, thus limiting the knowledge of the genes/proteins that are essential for ER pathogenesis and biology. As such, to identify proteins and/or mechanisms involved in ER virulence, we performed the first exhaustive comparative proteomic analysis between a virulent strain (ERGvir) and its high-passaged attenuated strain (ERGatt). Despite their different behaviors *in vivo* and *in vitro*, our results from 1DE-nanoLC-MS/MS showed that ERGvir and ERGatt share 80% of their proteins; this core proteome includes chaperones, proteins involved in metabolism, protein-DNA-RNA biosynthesis and processing, and bacterial effectors. Conventional 2DE revealed that 85% of the identified proteins are proteoforms, suggesting that post-translational modifications (namely glycosylation) are important in ER biology. Strain-specific proteins were also identified: while ERGatt has an increased number and overexpression of proteins involved in cell division, metabolism, transport and protein processing, ERGvir shows an overexpression of proteins and proteoforms (DIGE experiments) involved in pathogenesis such as Lpd, AnkA, VirB9 and B10, providing molecular evidence for its increased virulence *in vivo* and *in vitro*. Overall, our work reveals that ERGvir and ERGatt proteomes are streamlined to fulfill their biological function (maximum virulence for ERGvir and replicative capacity for ERGatt), and we provide both pioneering data and novel insights into the pathogenesis of this obligate intracellular bacterium.

## Introduction

*Ehrlichia ruminantium* (ER), an obligate intracellular bacterium of the order *Rickettsiales*, causes heartwater, a fatal and economically important disease of domestic and wild ruminants. This tick-borne disease occurs throughout sub-Saharan Africa and some islands in the Indian Ocean and the Caribbean, from where it threatens to invade the Americas, posing a serious threat to the livestock industry [1].

Several candidate vaccines such as inactivated, attenuated, DNA and subunit vaccines are available [2–6], but the development of a fully effective vaccine has been hindered by the difficulty of identifying protective antigens. This is due to the limited knowledge of the protective immune responses against heartwater and the lack of accurate *in vitro* antigen screening tests. Moreover, ER has a high antigenic diversity inducing low cross-protection between strains [1,7–12]. Several virulent ER strains have been reported [7–12] and at the moment, only two strains have been recognized as being attenuated by serial passage in bovine endothelial cells or canine macrophages: Senegal (from West Africa [13]) and Welgevonden (from South Africa [4]), respectively. A third one, Gardel strain (originated from Guadeloupe, French West Indies) has been suggested to be attenuated after roughly 200 passages in host endothelial cells [14] but no *in vivo* nor *in vitro* results have been so far published. Despite the recent advances in the study of ER biology [15–19], the lack of genetic tools for ER manipulation hampers knowledge of the genes that are actually expressed in live bacteria, namely those associated with ER virulence and attenuation.

Disparity in the pathogenicity or other biological aspects observed between strains can be related to important differences at the genomic level [20–22]. In ER, only 64% of the genome is predicted to be coding sequence (CDS), encoding 950 proteins and 39 stable RNA species [23]; 30% of these CDSs have unknown function. Genome analysis also indicates the presence of several classes of predictive virulence factors, such as genes/proteins involved in secretion and/or the trafficking of molecules between the pathogen and host cells, or evasion and/or modulation of the host immune system [16,17,24,25]. Still, comparative genomic analyses between the genomes of Gardel and Welgevonden strains indicate few genomic differences [25]. The gain of pathogenicity in bacteria can result from horizontal gene transfer, either directly or through mobile genetic elements [26]. In the case of an intracellular parasite such as ER, only one species of intracellular parasite inhabits a host cell, which restricts the parasite’s access to new genes, so few genes acquired by horizontal gene transfer are to be expected [24].

In this context, it can be assumed that differences between ER virulent and attenuated strains might not be predominantly at the genomic level but may be associated with variations in expression levels of genes/proteins encoding virulence factors, as previously observed for *Mycoplasma hyopneumoniae* [20].

Recent transcriptomic studies of ERGvir during its development cycle revealed that the extracellular and infectious form of the bacterium (elementary bodies, EBs) presented an overexpression of DksA [17], a transcription factor known to be related to virulence in other bacteria [27,28]. Preliminary proteomic studies on EBs also highlighted the expression of proteins related to virulence namely from the Type IV secretion system (T4SS, VirB9 and VirB11), and T4SS effectors AnkA, DksA and ElbB [29,30]. Still, no transcriptomic or proteomic studies comparing virulent and attenuated *ER* strains have been published. In fact, global “omics” studies on *Rickettsiales* species comparing virulent and avirulent strains are so far only available for *Rickettsia prowazekii* [22,31,32] and for *Rickettsia peacockii* [33].

In this study, we first confirmed the attenuation of Gardel after 200 passages *in vivo* and characterized ERGatt growth kinetics *in vitro*. Afterwards, we compared the proteomes of the ERGvir and ERGatt strains using gel-based separation approaches associated with MALDI-TOF/TOF mass spectrometry. Proteome analysis using 1DE-nLC-MS/MS revealed that both strains share 80% of their proteins (292 non-redundant protein), which represent approximately 31% (292/950) of the genome coding capacity of this bacterium. Surprisingly, 85% of the proteins identified by conventional 2DE electrophoresis are proteoforms. We also performed a quantitative proteomic analysis using DIGE to assess differentially expressed proteins between the two strains. This study is the first comprehensive and comparative proteomic analysis of virulent and attenuated ER strains and it provides an important proteomic basis for ER pathogenesis and suggests an important role of proteoforms in ER biology.

## Materials and Methods

### Experimental Design and Statistical Analyses

For the 1DE-nanoLC experiments and conventional 2DE gel analyses, three independent biological replicates per strain were used. Results from 1-DE-nanoLC-MS/MS experiments were analysed using PEAKS search engine tool (PEAKS Studio 5.3; Bioinformatics Solutions Inc., Waterloo, ON, Canada) [34], combining three search engines MASCOT, X!Tandem and Peaks DB. Results from conventional 2DE gels were analyzed and compiled using MDM software [35]. For DIGE experiments, four independent biological replicates per strain were used; image analyses were performed using Progenesis SameSpots v3.0 software and spot-normalized volume was used to select statistically significant (fold-change, ANOVA, false discovery rate and power value) differentiated spots between ERG strains analyzed in the experiment. All these parameters are described below in more detail.

### *E*. *ruminantium* cultivation and growth kinetics

Infected blood from ERG-infected goats was loaded on finite cultures of bovine aortic endothelial cells (BAE) to isolate the ERGardel virulent strain (ERGvir). ERGvir was then routinely propagated in BAE as described elsewhere [36,37]; ERGvir samples from passages up to passage 44 were used throughout this study, as they have been proven to be highly infectious in previous studies [38]. In order to obtain an attenuated ERG strain, ERGvir was cultivated over 230 passages in BAE cells, as suggested by Martinez (1987)[14]. When 80% cell lysis was observed (at 120hpi for ERGvir and 96hpi for ERGatt, S1 Fig), supernatant and cellular debris containing infectious ER elementary bodies were harvested and then used to (i) infect a freshly confluent monolayer or (ii) to be purified using a multistep centrifugation methodology [39]. Purified ERs were stored in SPG [39] at −80°C with a “Complete EDTA-free” anti-protease cocktail (Roche, Germany) prior to proteomic analysis. Prior to *in vivo* assays, purified ER were stored in SPG in liquid nitrogen [38].

Growth of ERGvir and ERGatt was monitored by phase-contrast light microscopy and quantified as previously described [17].

### Animal infection studies

To evaluate the virulent and attenuated status of ERGvir and ERGatt respectively, *in vivo* experiments were performed using naive goats from Les Saintes Island (Guadeloupe, FWI), a heartwater-free region. The ER inoculum was thawed and diluted in cold culture medium for immediate use on animals. Two goats (0541 and 0614) were infected intravenously with 72 x 10^4^ and 72 x 10^5^ viable elementary bodies from ERG passage 230 (ERGatt); the doses were calibrated as previously described [38] and correspond, respectively, to 2.4 and 24 times the lethal dose for the ERGvir strain [38–40]. As a positive control, one goat (0212) was infected with 72 x 10^4^ viable ERG passage 27. This part of the study was conducted in 2011–2012 according to internationally approved World Organization for Animal Health (OIE) standards, and CIRAD institution was authorized by the director of the veterinary services of Guadeloupe on behalf of the Prefect of Guadeloupe on August 2006 (authorization number: A-971-18-01). During these experiments, the intensity of the disease was monitored daily using a clinical reaction index, assigned to the different clinical symptoms [38,40]; daily clinical scores (S2 Fig) values correspond to the sum of clinical reaction indices *per* animal and *per* day. Goat 0212 (the positive control infected with ERGvir) was treated with antibiotics (tetracycline) at day 15 to prevent animal death (S2 Fig); no animals were sacrificed in this study.

### Protein extracts preparation

Purified ER were washed in TSB (Tris-sucrose buffer: 33mM Trizma, 250mM sucrose, pH 7.4) and centrifuged at 20,000 x g, for 30 minutes at 4°C. Pellets were snap frozen in liquid nitrogen and then dissolved in DIGE solubilization buffer (CHAPS (4%, w/v), urea (7M), thiourea (2M), Trizma (30mM)) using a vortex. Four cycles of freeze and thaw in liquid nitrogen were performed and samples were sonicated (using a microtip) on ice for 30 seconds (10% amplitude, 1 second ON, 0.5 second OFF) (Branson, USA). Samples were then centrifuged at 20,000 x g for 30 min at 4°C to pellet contaminants. Protein in the supernatant was then quantified using a 2D Quant kit (GE Healthcare, Sweden).

### 1DE-nanoLC experiments

#### 1D-SDS-PAGE

20 μg of ERGvir and ERGatt protein samples were loaded on pre-cast NuPage 4–12% Novex Tris-Glycine Gels, as described elsewhere [29]. Briefly, samples were solubilized in Laemmli buffer, under reducing conditions, and subjected to electrophoresis for 35 minutes in NuPage MES Buffer (all from Invitrogen, UK). Each gel included pre-stained molecular mass markers (SeeBlue 2, Invitrogen, UK). Proteins were stained with Coomassie/Colloidal Brilliant Blue R-250 and 14 gel bands were cut across the three ERGvir and three ERGatt biological replicates lanes (S3 Fig).

#### In-gel digestion

Each band was excised into smaller pieces, placed into a 1.5mL micro-centrifuge tube and washed with 100μL of MilliQ water twice for 15 min. Gel pieces were washed three times each with 50% acetonitrile and water and the samples were reduced at 56°C for 45 min with 10mM DTT in 100mM NH_4_HCO_3_ and alkylated for 30 min at RT with 55mM iodoacetamide in 100mM NH4HCO3. Gel pieces were then dehydrated twice with 100% acetonitrile for 15 min. Following drying in a SpeedVac, the gel pieces were mixed with 6.7μg/mL of trypsin (Promega, Madison, WI, USA) in 50mM NH4HCO3 and incubated on ice for 45 min. Tryptic digestion was carried out at 37°C overnight and stopped with 0.5% formic acid (Sigma). Tryptic peptides were extracted from the gel using 50% acetonitrile and water. Following 20 min of vortexing, the supernatant was collected and saved. The extraction was repeated once and all of the supernatants were combined. After evaporation of acetonitrile and water in a SpeedVac, the samples were dissolved in water with 0.1%AF (12μl) and loaded onto the Thermoplate for chromatographic separation [41].

#### NanoLC experiments

Chromatographic peptide separation was performed on a Thermo EASY-nLC 1000 with a pre-column Acclaim PepMap 100 C18 (75 μm x 2 cm) used as peptrap and an Acclaim PepMap RSLC C18 (50μm x 15cm) as the chromatographic separation column, as previously described [41]. Briefly, a chromatographic gradient was established using mixed volumes of 0.1% formic acid in water (buffer A) and 0.1% formic acid in ACN (buffer B, all LC-MS grade, from MERCK); the peptides were eluted in 5–40% buffer A for 40min according to their hydrophilic/hydrophobic properties. Peptide fractions were spotted onto MALDI plates with alpha-Cyano-4-hydroxycinnamic acid (5mg/mL, Sigma) at a constant rate of 2ml/min using a micro-spotter (Sunchrom, Germany). Maldi plates were then analyzed on a 4800 MALDI TOF/TOF instrument (Applied Biosystems, MA, USA) with 4000 series explorer v3.5 software.

### Conventional 2DE

#### Isoelectric focussing (IEF)

Protein separation of three biological replicates *per* strain was performed using 600μg of protein. This amount of protein was diluted in DIGE buffer (as described above and supplemented with IPG buffer pH 3–10 NL (0.8%, v/v) and 60mM DTT) to a final volume of 450μl. IEF was then performed using the IPGphor system (using cup loading and manifold) and 24cm immobiline drystrips with a non-linear pH gradient from 3 to 10 (all from GE Healthcare, Sweden). The protocol consisted of a sequence of 7 steps as follows: rehydration of the strips (50μA/strip at 20°C) was carried out for 12 h at 30V, followed by a step-and-hold running condition at 100Vh (3h), step from 300V to 600Vhr, step from 500V to 500Vhr, gradient at 3500V (2h), step from 3500V to 7000Vhr, gradient at 10000Vh (3h), and a final step at 10000Vh (5h), with a total of 77,7kVh.

#### SDS-PAGE

After IEF, the IPG strips were equilibrated: first the samples were reduced in equilibration buffer (50mM Tris pH 8.8, 6M urea, 30% (v/v) glycerol and 2% (w/v) SDS) supplemented with DTT (10mg/ml), followed by alkylation in equilibration buffer supplemented with iodocetamide (25 mg/ml). Each equilibration step lasted 15 min under slow agitation at room temperature [42]. Electrophoresis was then performed as previously described [29]. The IPG strips were then embedded in a precast gel (GE Healthcare, Sweden) and sealed into place using 0.5% (w/v) agarose sealing solution. The SDS-PAGE was performed using an Ettan six DALT system (GE Healthcare, Sweden) with a discontinuous buffer system of SDS electrophoresis buffer (25 mM Tris-HCl, pH 8.3, 192 mM glycine, 0.1% (w/v) SDS) in the bottom chamber and SDS electrophoresis buffer (50 mM Tris-HC pH 8.3, 384 mM gycine, 0,2% (w/v) SDS) in the top chamber overnight at 150 V, 12 mA/gel, 2 W/gel. Gels were stained using Colloidal Coomassie Blue, according to methodology described by Neuhoff et al. [43]. Briefly, gels were stained for 48h and subsequently washed three times in double distilled water. Gels were stored at 4°C in a 20% (w/v) ammonium sulphate solution until image acquiring and spot excision. Digital images of the gels were acquired using a laser-based scanner FLA-5100 (Fuji Inc., Japan). Three biological replicates were used per strain.

### 2D-DIGE

Each protein sample (50 μg) was labeled with 400 pmol of Cy3 or Cy5, and Cy2 was used as an internal calibrator. After incubating on ice for 30 min in the dark, the labelling reaction was stopped with 10 mM lysine. For each gel, Cy3- and Cy5-labeled proteins were mixed with 130μL rehydration buffer (7M urea, 2M thiourea, 2% (w/v) CHAPS, 130mM DTT, 2% IPG buffer pH 3–10). The labeled protein mixture was applied to Immobiline DryStrip strips (24cm, pH 3-10NL; GE Healthcare). Isoelectric focusing (IEF) and SDS-PAGE was performed as described above. Gel images were acquired on a laser-based scanner FLA-5100 (Fuji Inc., Japan) using 532nm and 635nm excitation lasers (DGR1double filter) for Cy3 and Cy5 respectively, and 473nm excitation laser (LPB filter) for Cy2 under Image Reader FLA 500 version 1.0 (FujiFilm). The gels were scanned using low-fluorescence glass plates at a resolution of 100μm. Four biological replicates per strain were used.

### Image analysis

Coomassie and DIGE images were analyzed using the Progenesis SameSpots v3.0 software (Non-linear Dynamics, Newcastle, UK). First, images were aligned. Next, prominent spots were used to manually assign vectors to digitized images within each gel and then the automatic vector tool was used to add additional vectors, which were manually revised and edited for correction if necessary. These vectors were used to warp and align gel images with a reference image of one internal standard across and within each gel. After automatic spot detection, spots were manually revised with editing tools for correct detection.

For DIGE analysis, gel groups were established according to the experimental design and spot-normalized volume was used to select statistically significant (fold-change, ANOVA, false discovery rate and power value) differentiated spots between ERG strains analyzed in the experiment. A value of 1.5-fold increase or decrease was used as a cut-off and statistically significant differences in spot intensities were identified using a Student's *t*-test with *p*<0.05 (with visual inspection of the results).

A protein spot list was generated and the spots with significant changes were excised from the Coomassie gels. Excision of protein spots was done as follows: with a set of paper reference circles attached to each side of the glass plate, the ordinance information for each protein of interest was translated and transferred to an automatic spot picker (GE Ettan Spot Handling Work Station, Sweden) through the pick list. Spots then were excised by the picker and transferred to a 96-well collecting plate containing 120μL of a 10% ethanol solution. Plates with excised spots were stored at -20°C until further use.

### In-gel digestion for 2DE-spots

The proteins of interest were digested into their component peptides with trypsin and eluted from the gel plugs, as described above. Briefly, spots were excised, destained, reduced with DTT, alkylated with iodoacetamide, and dried in a speedvac. Gel pieces were rehydrated with digestion buffer (50 mM NH_4_HCO_3_) containing trypsin (6.7 ng/μl) (Promega, Madison, WI, USA) and incubated overnight at 37°C. The buffered peptides were acidified with formic acid, desalted and concentrated using homemade reversed phase microcolumns (POROS R2, Applied Biosystems, Foster City, CA, USA). The peptides were eluted onto a MALDI plate using a matrix solution that contained 5 mg/ml α-cyano-4- hydroxycinnamic acid dissolved in 50% (v/v) ACN/0.1% (v/v) formic acid.

### Protein identification by MALDI-TOF/TOF

Protein identification was done on a 4800 MALDI-TOF/TOF instrument (Applied Biosystems, Framingham, MA, USA) with 4000 series explorer v3.5 software in both MS and MS/MS mode. Each MS spectrum was obtained in a result independent acquisition mode with a total of 800 laser shots per spectrum, with internal calibration using Angiotensin II (1046.2 Da), Angiotensin I (1296.5 Da), Neurotensin (1672.9 Da), ACTH [1–17] (2093.5 Da), ACTH [18–39] (2465.7 Da) (PepMix1, from Laserbio labs). Fifteen s/n best precursors from each MS spectrum were selected for MS/MS analysis, starting with the most intense peak and ending with the least intense peak. MS/MS analyses were performed using CID (Collision Induced Dissociation) assisted with air, using a collision energy of 1 kV and a gas pressure of 1 × 10^-6t^ Torr. A total of 1400 laser shots were collected for each MS/MS spectrum with the peak detection minimum signal to noise (S/N) setting at 20.

The MS/MS spectra obtained for the samples processed by 1DE-nanoLC were searched against the database search using PEAKS search engine tool (PEAKS Studio 5.3; Bioinformatics Solutions Inc., Waterloo, ON, Canada) [34], combining 3 search engines MASCOT, X!Tandem and Peaks DB (S1, S2 and S3 Tables). The search parameters for the MS/MS spectra are presented in S1 File.

For protein identification in 2D gels, Mascot Generic Files combining MS and MS/MS spectra were automatically created and used to interrogate a non-redundant protein database using a local version of Mascot v2.2 from Matrix Science through the Global Protein Server (GPS) v3.6 (Applied Biosystems). The search parameters for the MS/MS spectra were described in S1 File. MDM [41] was used to organize Mascot files into Tables (S4 and S5 Tables).

### Protein functional annotation

The identified proteins were classified according to Collins et al [24], Frutos et al ([25], Moumene et al [19], Meyer et al [16], the Clusters of Orthologous genes (COG) functional categories [44,45], the online pathway tools on the Kyoto Encyclopedia of Genes and Genomes web site (KEGG, (http://www.genome.jp/kegg-bin/show_pathway?org_name=erg&mapno=01100&mapscale=0.35&show_description=hide&show_module_list) [46] and Uniprot database (http://www.uniprot.org/proteomes/UP000000533). For proteins with no assigned function, homology searches were performed using the BlastP program against all non-redundant protein sequences deposited in the NCBI database http://blast.ncbi.nlm.nih.gov/Blast.cgi and the Uniprot database website. Protein hits were considered to be significant when their *e*-value was below 10^−5^.

### *In silico* PTM analysis

An adapted version of the QuickMod software [47] was used to search for unidentified modifications in the MS/MS data of MAP1 (ERGA_CDS_09160) and the Porin_05140 (ERGA_CDS_05140) protein spots. First, all MAP1 or Porin_05140 spectra were clustered in order to increase the spectrum quality by forming consensus spectra. Then we searched all MS/MS spectra against all consensus spectra in Open Modification Search (OMS) mode [48]. This approach relies on the alignment of modified query spectra to database spectra, where some fragment peaks are shifted in accordance with the mass of the modification(s) [49].

## Results

### Validation of the attenuated status of ERGatt strain

ERGvir has previously been shown to be highly virulent *in vivo*, leading to animal death, unless it is treated with antibiotics (tetracycline) [1,38–40]. Its growth kinetics *in vitro* have also been previously described [37], presenting a complete life cycle within 120 hours post-infection (hpi). Herein, we showed for the first time that ERG virulence was attenuated after 230 passages *in vitro*. Indeed, naive goats injected with high doses of ERGp230 (goats 0541 and 0614) survived the infection with low clinical signs (only mild hyperthermia occurred during 6 days). The goat infected with a lethal dose of ERGvir (goat 0212) presented strong clinical symptoms (high fever and neurological symptoms associated in general with fatality) and was treated with antibiotics to avoid animal death (S2 Fig). Another independent experiment with 4 goats infected with ERGatt resulted in similar results, with low clinical symptoms for naive goats infected with ERGatt (data not shown). *In vitro*, we observed that ERGatt presented a shorter life cycle of 96 hpi instead of 120hpi for ERGvir (S1A Fig). Interestingly, we could use a high multiplicity of infection for ERGatt sub-culturing without any cytotoxic effect on host cells (data not shown). On the contrary, for ERGvir, the inocula should not be above 400 ER/host cell (1/20 of the starting volume) as it can lead to cell death at the time of infection [50]. Moreover, a higher number of morula *per* host cell is also observed in ERGatt-infected cells (S1B Fig).

### General features of the *E*.*ruminantium* Gardel proteome

**Fig 1:**
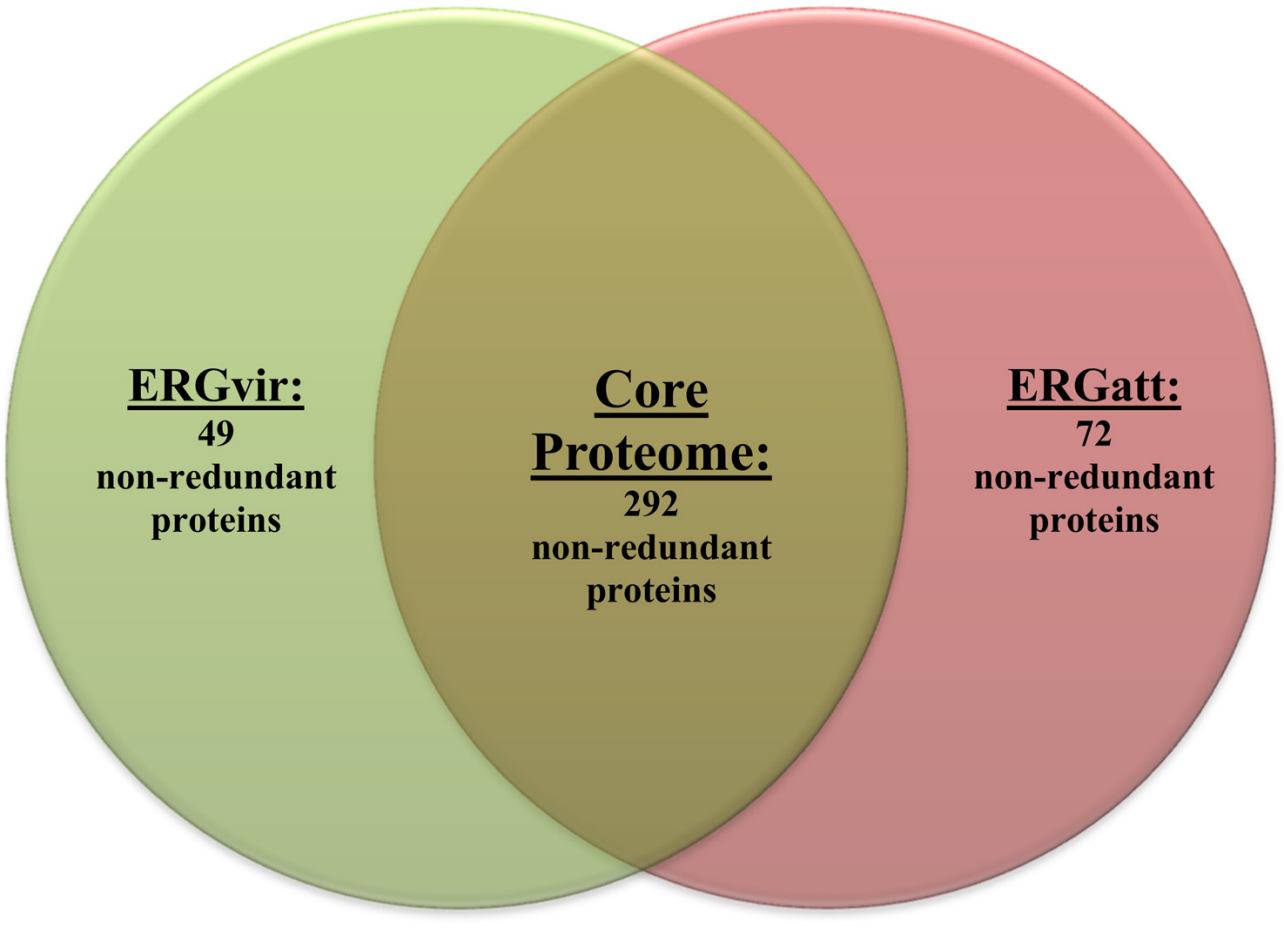
Venn diagram representing the number of non-redundant proteins identified in ERGvir and ERGatt and in both (core proteome) by 1DE-nanoLC-MALDI-TOF/TOF analysis. Three biological replicates per strain were independently used and the results analyzed with Peaks software (using using simultaneously Peaks DB, MASCOT and X!Tandem algorithms).

Whole proteome analysis of ERGvir and ERGatt strains using 1DE-nanoLC-MALDI-TOF/TOF and Peaks software analysis resulted in the identification of 341 and 364 non-redundant proteins, respectively. Of these, 292 proteins (31% (292/950) of the CDS in ERG genome [25] (S1 Table, Fig 1) were found to be common between ERGvir and ERGatt, which corresponds to approximately 80% of the identified ERG proteome. This low variability in the identified proteins confirms the few observed differences in the SDS-PAGE migration pattern (S3 Fig). ERGvir and ERGatt expressed 49 and 72 strain-specific proteins respectively (Fig 1, S2 and S3 Tables).

To complement the data from 1DE-nanoLC, we also used conventional bidimensional electrophoresis (2DE). S4 Fig is a representative example of a 2DE gel obtained for the three biological replicates used within this study. It shows that proteins exhibit isoelectric points (pI) ranging from 4 to 10 with most of them having an acidic pI and molecular mass (MM) range from 10 to 157 kDa (S4 Fig). Of all spots analyzed per gel, only about 50% were identified as ER proteins (S4 Table), the others being proteins of host cell origin (data not shown). The 2DE data were generally in agreement with the data obtained by 1DE-nanoLC for both strains, although there were some proteins (such as AnkC, PurE, IhfA, Def, PdxJ, ThiC, RibH, Dut, Rsfs, PpnK, PdhA, GatC and Rho) that were exclusively detected by 2DE and DIGE (S4 and S5 Tables). The sequence coverage of the identified proteins ranged from 1.1% (spot 506, protein PutA) to 78.5% (spot 2468, protein TsaA) (S4 and S5 Tables). Interestingly, we also observed that 71 spots (approx 15% (71/483 spots) of the whole conventional 2DE proteome) corresponded to non-redundant ERG proteins while all the other proteins were found in multiple spots (from 2 to 36); this suggests that ER single genes resulted in several protein molecules (“proteoforms”) due to genetic variation or post-translational modifications [51] (Table 1). MAP1 (ERGA_CDS_09160, with 36 spots) and Porin_05140 (ERGA_CDS_05140, with 27 spots) were found to be the proteins with the highest number of proteoforms in the ERG proteome map. In an attempt to identify the PTMs associated to each protein spot, we used an adapted version of the QuickMod software [47]. The analyses revealed many modification mass shifts of +14Da, +16Da, +28Da, +48Da, +91Da and +283Da between different MAP1 spots, as well as +14Da, +15Da, +16Da, +91Da and +283Da between different Porin_5140 spots. These mass shifts could correspond to methylation (+14Da, +28Da), oxidation (+16Da) and GlcNAc (+283Da) or sequence variations. The best OMS scores revealed that some proteoforms of MAP1 (spots 2372, 3464 and 2581, S4 Fig) and Porin (spots 1765, 1815, 1885 and 1814, S4 Fig) correspond to N-glycosylated proteins.

**Table 1:** Maximum number of spots per protein (proteoforms) detected by conventional 2DE-MALDI-TOF/TOF (N = 6).

Protein name	Maximum nb of proteins spots detected in 2DE gels (N = 6)
Map1	36
Porin_05140	27
Tuf1/Tuf2	15
GroES	12
TsaA, DnaK, ClpB, GroEL, Q5FGU8	9
ArgD, Pal, GlyA	8
AtpA,Q5FH79	7
RpoC, Bcp, FtsZ	6
Q5FGD3, VirB9	5
DapE, ClpX, HtpG, Ssb, IscS, Q5FFV7, PepA, SdhB, Q5FGB7, TolC	4
RibB, GrpE, Tme, SucC, Tig, RpoD, ArgG, AtpD, SodB, FolP, FolK, PpdK, SdhA, SecB, PurD, Q5FGI8, Ndk, Map1-1, Map1+1, Q5FGQ4, Q5FGQ2, Def, RibB, RpoA, SucB	3
VirB11, GlnA, PdhB, SucD, FusA, RplK, RplA, RplL, FabF,DapA, Q5FHE9, HupB, GatA, Mdh, HscA, ArgB, NusA, FumC, FolD, RpsF, Trx, GltX1, Rho, Map1-14, ElbB, Q5FF93, Efp, GyrB, Icd, Lpd, Pnp, PurH, PutA	2

### Analysis of the functional distribution of the proteome

**Fig 2:**
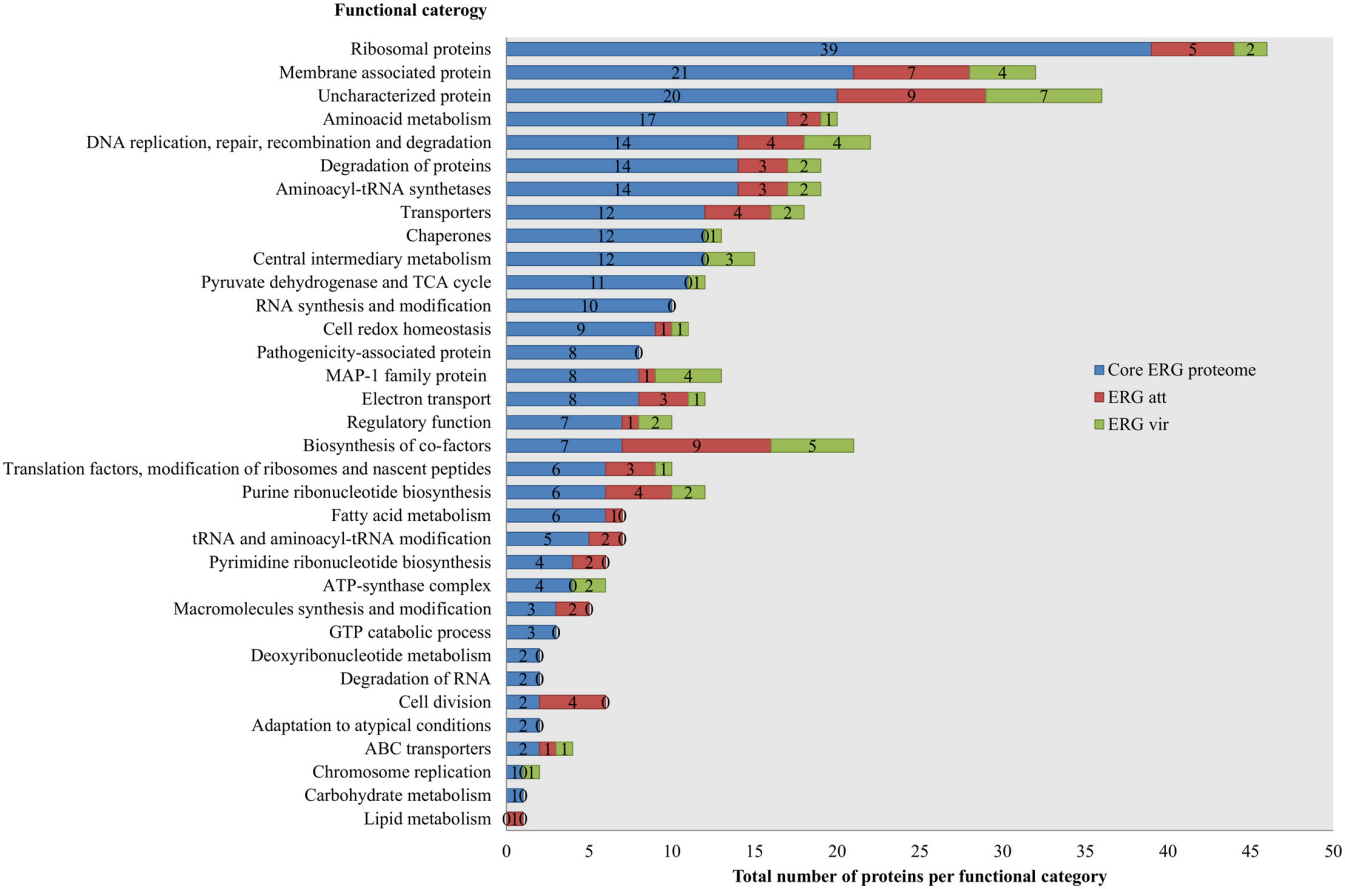
Functional distribution of the identified proteins in ERGvir (green) and ERGatt (red) by 1DE- nanoLC-MALDI-TOF/TOF and after Peaks software analysis (using simultaneously Peaks DB, MASCOT and X!Tandem algorithms). The proteins constituting the core proteome are depicted in blue. The number of identified proteins associated with each COG functional category is shown in the X axis (total number) and in the graph bars (number per strain, n = 3).

The core proteome of ERG consists of 292 proteins common to both strains, as detected by 1DE-nanoLC whose functional distribution is represented in Fig 2 (blue color code). As predicted from the ER genome [24,25] and previous studies of ER proteomics and transcriptomics [18], ER has a high number of uncharacterized proteins (30% of CDS). We identified bacterial effectors homologous to AnkA, B and C, Ats-1, ApxR and a Bax-1 related protein by manual reannotation of uncharacterized proteins. Proteins exclusively expressed in ERGvir or ERGatt ERG reflect the proteome diversity and the proteins that may be involved in the transition from virulence to attenuation. We observed that ERGvir has a higher number of proteins related to the MAP-1 family proteins but also a higher number related to central intermediary metabolism, regulatory function, the ATP-synthase complex, chaperones, pyruvate dehydrogenase, the TCA cycle and accurate chromosome replication (Fig 2, S2 Table). ERGatt expresses a higher of number of proteins involved in the biosynthesis of co-factors, cell division, metabolism (lipid, fatty acid, aminoacids), membrane-associated proteins (including transporters), DNA-RNA-protein synthesis and processing (Fig 2, S3 Table).

### Alterations in protein abundance between virulent and attenuated ERG strains

**Fig 3:**
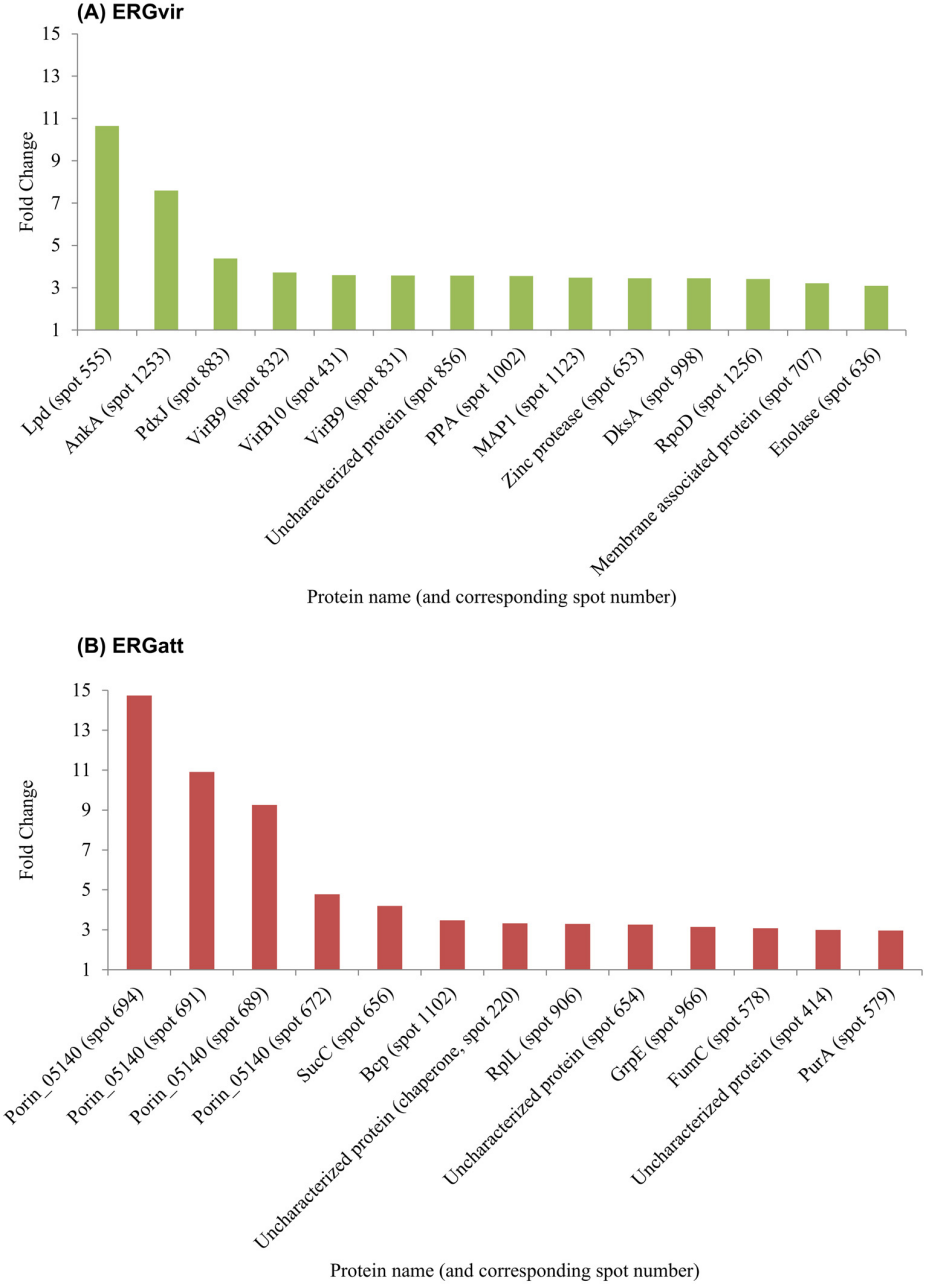
Proteins overexpressed (fold change ≥ 3) in ERGvir (A) and ERGatt (B) according to DIGE analyses and MALDI-TOF/TOF.

To quantitatively assess proteins differentially expressed between ERGvir and ERGatt strains we used a DIGE strategy. Image analysis with Samespots software, revealed that the expression levels of 117 proteins were found to be significantly differentially expressed between ERGvir and ERGatt (p<0.05), 67 being overexpressed in ERGvir and 48 in ERGatt (S5 Fig and S5 Table). In ERGvir, some of the proteins with higher fold-change are known virulence factors in other bacteria, or related to cell redox homeostasis (such as AnkA and one proteoform of Lpd) (Fig 3A). Interestingly, amongst the proteins overexpressed by ERGatt, there are 4 proteoforms of the Porin_05140 (Fig 3B).

## Discussion

ER was first identified more than 90 years ago but little data on its biology and pathogenesis are currently available. The lack of genetic manipulation techniques for ER has hampered an understanding of the role of numerous uncharacterized genes and the comprehension of virulence/attenuation mechanisms.

**Fig 4:**
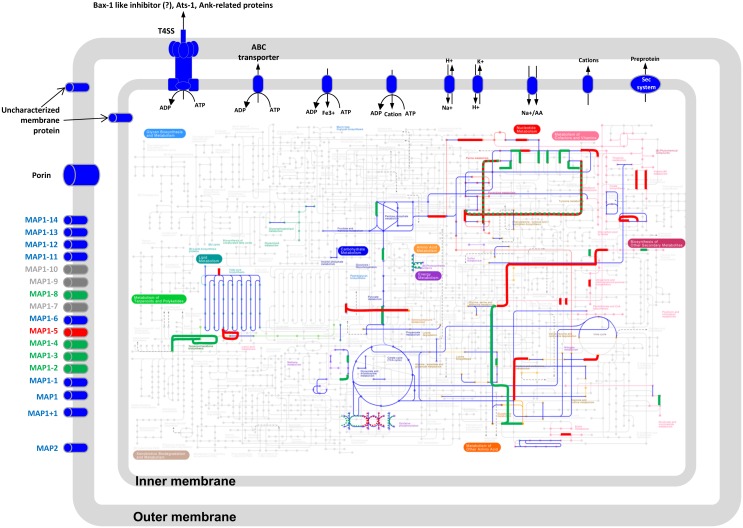
Schematic overview of metabolic pathways and membrane proteins found in ERG, based on genomic (KEGG database) and proteomic information obtained in this work. Nodes correspond to substrates and edges to enzymatic reactions and the 11 major metabolic pathways are color-coded (for example, light orange for amino acid metabolism). The proteins that were identified on both ERGvir and ERGatt are indicated in dark blue, the proteins and pathways found exclusively in ERGatt are in red and those detected only in ERGvir are highlighted in green. The dashed lines correspond to metabolic pathways found in both strains.

*In vitro* and *in vivo* infection assays revealed that the ER virulent and attenuated Gardel strains behave differently: the virulent strain is highly pathogenic to goats and can be toxic to host cells *in vitro* immediately after infection [50] while the attenuated strain undergoes a shorter life cycle and does not induce death in goats. The aim of our work was to identify virulence and attenuation-associated proteins and find proteins and/or biological processes that may have impacted the biology of ER during its long term-passaging *in vitro*, contributing to the attenuation process (Fig 4).

### General features of *Ehrlichia ruminantium* Gardel proteomes

Despite the *in vivo* and *in vitro* differences, ERGvir and ERGatt share 80% of the identified proteins, constituting a core proteome (Figs 1 and 2, S1 Table). This is in agreement with other proteome studies comparing bacterial strains, that have regularly found the core proteome to consist of 70 to 90% of the identified proteins [29,52].

The ERG core proteome includes proteins from several functional categories, namely proteins involved in DNA-RNA-protein processing; aminoacid/carbohydrates biosynthesis, metabolism and transport; energy production; intracellular trafficking and secretion and proteins involved in homeostasis control (Fig 2). This global set of proteins is essential for the biology of ERG. These types of proteins were also shown to be crucial for other *Rickettsiales* species such as *Anaplasma phagocytophilum*, *Anaplasma marginale* and *Ehrlichia chaffensis* [18,53]. Proteins with uncharacterized function were one of the major groups of protein detected due to the lack of homology with other organisms. As these proteins are within the core proteome they must also be essential for ER biology.

Whole comparative proteomic profiling between the two Gardel strains also allowed us to detect strain-specific proteins, which might be related to virulent and attenuated status. For instance, ERGvir expresses the well conserved ParA/ParB system [54], which is known to guarantee an accurate partitioning of chromosomes between bacterial daughter cells prior to cell division. The ParA protein was not detected in ERGatt. We thus suggest that long-term passaging of ERGvir *in vitro* could have resulted in the loss of ParA protein affecting chromosome segregation and eventually bacteria growth rate, as previously observed for *Pseudomonas aeruginosa* [55]. Additionally, ERGatt was found to have a higher number of proteins involved in cellular replication (ERGA_CDS_06380, ERGA_CDS_06680, ERGA_CDS_0882, ERGA_CDS_08930) which supports its increased growth rate *in vitro* compared to ERGvir. On the other hand, quantitative differential proteomics indicate that differences in virulence might not only be associated with strain-specific proteins (S2 and S3 Tables) but also related to differential regulation of common biochemical processes (Fig 3, S5 Table). These topics will be discussed in more detail below.

An interesting feature of the ER proteomes is the high number of protein proteoforms expressed in both strains. In a previous work performed by our group with ERGvir [29], we discovered that 25% of the proteins identified were proteoforms. Herein, after optimizing protein extract preparation and 2DE conditions we found that up to 85% of proteins were proteoforms. Moreover, our results in DIGE experiments clearly revealed that different proteoforms are differently expressed between virulent and attenuated ER strains. Both results clearly indicate the biological importance of PTMs in ER, although we do not know yet their real impact on the bacterium biology and pathogenesis. In pathogenic bacteria, post-translationally modified proteins can promote bacterial survival, replication, and evasion from the host immune system [56]. For instance, PTMs such as lipoylation, glycosylation, phosphorylation and SUMOylation can have a high impact on host immune modulation during *Ehrlichia muris* [57], *Ehrlichia chaffeensis* [58] and *Anaplasma phagocytophilum* [59] infections and, multimethylation can lead to different levels of virulence in *R*. *prowazekii* strains [22,60]. *In silico* analysis of MS/MS data from two major proteins (MAP1 and Porin_05140) revealed that some ER protein proteoforms have N-glycosylated moieties. This corroborates with the preliminary results obtained by Postigo and co-workers [30] regarding the glycosylation of MAP1 protein proteoforms. Preliminary assays of ER PTM mapping performed by our group revealed that more than 50% of ER proteome is composed of glycoproteins [61].

### *Ehrlichia ruminantium* basic metabolic activities

Functional studies of obligate intracellular bacteria from the *Rickettsiales* order reveal the presence of several genes/proteins involved in (i) energy production and conversion and (ii) the transport and metabolism of nucleotides, aminoacids, inorganic ions, carbohydrate and co-enzymes [18]. Our results summarized in Fig 4 are in general agreement with these findings.

More specifically, ERGvir and ERGatt strains express enzymes involved in the Embden-Meyerhof-Parnas pathway, aminoacid metabolism, including one Proline/Betaine transporter for aminoacids (Fig 4). All predicted enzymes of the TCA cycle [24] were also found (Fig 4). We also detected the proteins involved in a partial gluconeogenesis pathway and a complete non-oxidative pentose-phosphate pathway (Fig 4).

In the virulent strain, we detected a higher number of proteins involved in energy conversion and production. This energy could be use to fuel specific processes, namely those involved in virulence. Interestingly, a higher number of proteins related to metabolism of lipids and amino acids, protein processing and biosynthesis of co-factors was detected in ERGatt (Fig 4); this could relate to a higher growth rate of ERGatt *in vitro*. This could also suggest that ERGatt is metabolically more efficient than ERGvir and that it might not need to compete with bovine endothelial host cells for essential vitamins and nucleotides, and may even supply them to the host cells. Similar processes have been proposed to occur between the obligatory intracellular bacterium *Wigglesworthia glossinidia* and its insect host, tsetse fly [62] and for *Anaplasma phagocytophilum* and *Ehrlichia chaffeensis* [52].

### Expression of ERG membrane proteins

Membrane proteins are an important group of proteins as they perform a variety of functions vital to the survival of organisms (host invasion, transport, immune response, adhesion, etc). As predicted from the ER genome annotation [24,25] and previous proteomic analyses [19,29], ER has many membrane proteins. Here, we found 21 membrane proteins in the ERG core proteome, including the members from the Major Antigenic Protein 1 (MAP1) family proteins (Figs 2 and 4). Indeed, 8 out of the sixteen MAP1 paralogs (MAP1, MAP1+1, MAP1-13, MAP1-6, MAP1-1, MAP1-11, MAP1-12, MAP1-14) were detected in both ERG strains (Fig 4). Some of these proteins (MAP1, MAP1+1, MAP1-6, MAP1-13 and MAP1-14) were previously detected in host endothelial cell cultures [29,30]. Here we report for the first time that MAP1-1 is found in ER cultivated in BAE cells, and not only in tick cell lines as previously mentioned [30,63]. It has also been previously observed that the ER *map1* gene cluster can be differentially expressed according to the microenvironment (host-tick-extracellular milieu) but also according to the ER strain [63]. Our results identified a total of 12 MAP1 related proteins in ERGvir and nine MAP1 family proteins in ERGatt (Fig 4). Additionally, we found that some of these proteins have several proteoforms: 2 for MAP1-14, 3 for MAP1+1 and MAP1-1 and 36 for MAP1 (known to be the most abundant protein in ERG [29]). Although the role of MAP1-family proteins in ER biology has not yet been established, they are known to be highly immunogenic; we suggest that they could then be used by the virulent strain as bait to confound the host immune system. From the results presented above, it is clear that they must be relevant for ER biology and further investigation into their role is necessary.

We also detected the porin ERG_CDS_05140, which appears to be the second most abundant protein in ERG after MAP1. This protein was found in both strains but four proteoforms were mainly expressed in ERGatt. Although we do not know the role of this protein in ERG biology, we suggest that this porin could contribute to the exchange of small metabolites (sugar, aminoacids, etc) between ER and its environment/host cell and thereby contribute to the increased growth rate in ERGatt. Additional studies are currently being performed by our group to expand the knowledge of the function of this outer membrane protein.

Another interesting membrane protein common to both strains is ERGA_CDS_05800, which is homologous to the *A*.*phagocytophilum* invasin OmpA [64]. OmpA (outer membrane protein A) is conserved among most Gram-negative bacteria and is an important virulence factors for several Gram-negative pathogens [64–66]. It could be thus be used by both ERG strains during host cell invasion.

In ERGvir, we detected two additional proteins that could be used by this strain to invade the host cells: the enolase (ERGA_CDS_04960, Fig 3) and the protein ERGA_CDS_00060 which is homologous to *A*.*phagocytophilum* hemolysin (S2 Table). Enolase is a prototypic moonlighting protein in both prokaryotes and eukaryotes [67,68] and has been recently recognized to have a significant impact in a variety of pathophysiological processes. Hemolysins are membranolytic enzymes that form pores of varying diameters in the membrane of cells. This protein could thus aid ER invasion, and it has been previously observed in other bacterial infections [69–71]. Its presence uniquely in ERGvir could also support the cytotoxic effect in host cell cultivated *in vitro* culture conditions and its invasion capability *in vivo*.

### Proteins involved in *E*.*ruminantium*-host interactions

In order to be able to infect and successfully colonize the host, ER must rapidly modulate host cell gene transcription and function after adhesion/invasion. In this study, several transporters and molecules involved in ER-host cell cross talk were detected in both strains, including proteins from the Sec pathway (involved in both the secretion of unfolded proteins across the cytoplasmic membrane and the insertion of membrane proteins into the cytoplasmic membrane [72]) and also those related to the Type Four Secretion System (T4SS). The role of the T4SS in the pathogenicity or parasitic lifestyle of bacterial pathogens is well established, including in other *Rickettsiales* such as *A*. *marginale*, *E*. *canis* and *E*. *chaffeensis* [18,73–75]. Here, we detected 8 out the 10 “building blocks” of the ER T4SS [76] (VirB10, B11, B2, B4, B6, B8, B9 and VirD4, S1 Table). Manual reannotation of proteins allowed us to identify ER bacterial effectors homologous to *A*.*phagocytophilum* proteins, such as T4SS-secreted proteins AnkA (previously predicted in ER by S4TE software [16] and detected in ERGvir [29]), Ank–B-C, Ats-1 and AprX proteins. We also identified a BAX inhibitor (BI)-1 like protein. Ats-1 and BI-1 like protein are known to interfere with apoptosis [77], and ApxR and Ank-related proteins regulate gene expression [78–80].

Differential expression analyses using DIGE revealed that DksA (known as a virulent factor in *Salmonella typhimurium* [28] and enterohemorrhagic *Escherichia coli* [27]) and AnkA are overpexpressed in ERGvir (Fig 3A). As above mentioned, AnkA alters host cell gene expression; an increased level of this protein in ERGvir would facilitate host manipulation, interfering with cellular responses in inflammatory diseases [81]. VirB9 and B10 proteins were also found to be overexpressed in ERGvir (fold change > 3, Fig 3A). Increased amounts of these T4SS core complex building blocks could be used as a support for intracellular development and therefore contribute for increased virulence of the bacteria.

In the ERGatt proteome, we detected a Patatin like-protein (ERGA_CDS_01780, homologous to *E*.*chaffeensis* EchaDRAFT_0464). This intracellular cytotoxin is known to perturb membrane trafficking and modulate intracellular bacterial growth [82]; it could thus promote intracellular replication inside host cells, again contributing to the increased growth rate of ERGatt *in vitro*.

### Proteins involved in *E*.*ruminantium* survival in the extracellular milieu

During its period outside the host cells as an infectious elementary body, ER must interact with its surroundings and protect itself from adverse conditions. Two-Component Signal transduction systems (TCS) are known to be important for these interactions as they allow organisms (especially prokaryotes) to sense and respond to changes in many different environmental conditions [83,84]. They typically consist of a membrane-bound histidine kinase that senses a specific environmental stimulus and a corresponding response regulator that mediates the cellular response, mostly through differential expression of target genes. Several *Rickettsiales* (including ER) are known to have the genes coding for three histidine kinases (homologs of NtrY, PleC, and CckA) and their corresponding response regulators (homologs of NtrX, PleD, and CtrA) [83]. In both virulent and attenuated ERG strains, we detected the response regulators CtrA and PleD, while the sensor protein NtrY was only identified in ERGatt (S1–S3 Tables). In comparison, the three potential pairs of TCSs were detected in *E*.*chaffeensis* and *A*.*phagocytophilum* using double immunofluorescence labeling and Western blot analysis (using polyclonal antibodies) but only during their intracellular development, suggesting that TCSs might be active mostly during the bacterial intracellular development [83].

Several chaperones (such as DnaK, DnaJ, HslU, HslV, GroEL, GroES, and HtpG proteins) and other key proteins involved in cell homeostasis/oxidative stress response (such as PepA, ClpP, ClpB, DnaK, SurE, ElbB, TsaA) were also identified in both strains. Some of these proteins were previously detected in ERG [29] and in other *Rickettsiales* [53,85,86]. Interestingly, one of the overexpressed proteins in ERGvir is the protein Lpd (Fig 3A). Although it is generally considered to be involved in cell homeostasis, in *A*. *phagocytophilum*, this protein was found to act as an immunopathological molecule, affecting cytokine and chemokine production [87]. It is an important virulent factor in several bacteria such as *Mycobacterium tuberculosis* [88], *Mycoplasma gallisepticum* [89] and *Pseudomonas aeruginosa* [90]. As host endothelial cells are able to produce cytokines and chemokines, high Lpd expression could explain the strong clinical signs observed *in vivo* during the infection with ERGvir [39] and their eventual toxicity *in vitro* [50]. Apart from the proteins found in the core proteome, no additional known proteins related to pathogen-extracellular milieu interaction were detected specifically in ERGatt.

### From virulence to attenuation: what happens?

From all the points discussed above, we propose the following hypothesis for the conversion of ERGvir to ERGatt: *In vivo*, ERGvir needs to have the “tools” to protect itself from the external environment/immune system and rapidly infect new target cells. To avoid the dangers of the surroundings, ERGvir expresses not only a high number of chaperones and proteins to avoid oxidative stress, but also a higher number of MAP1-related proteins that induce a high but unprotective antibody response. To efficiently infect target cells, ERGvir expresses more proteins related to central intermediary metabolism to fuel the higher number of proteins related to virulence (AnkA, Hemolysin, Lpd, Enolase, VirB-proteins, etc). The high number of proteoforms would also provide an efficient strategy to perform the tasks required for infection and survival. Indeed, different proteoforms with slight difference in their PTM could “sidetrack” the immune system while others could be essential for ER pathogenesis. After successive long-term passaging *in vitro* with no major selective pressure from the immune system, ERGvir adapts itself to the less constraining *in vitro* culture conditions, and eventually suffers from mutations (eventually due to the loss of ParA protein). This adaptation could also be coupled to the overexpression of proteins related to cell division, biosynthesis of co-factors, electron transport, membrane associated proteins, ribosomal proteins, proteins associated to protein production and processing and transporters in ERGatt, all resulting in rapid bacterial replication. Interestingly, ERGatt has 4 major proteoforms of a porin that could also contribute to a more efficient metabolism. On the other hand, the disappearance or lower expression of virulence-associated proteins is also observed in ERGatt, which could explain why animals survive without major clinical signs when ERGatt is administrated.

## Conclusion

By establishing the most complete proteome profiling of two ER strains with different levels of virulence, we helped to answer some questions raised by ER genome sequences and to highlight the role and the structure of key proteins involved in ER survival, infectivity and attenuation. This is of major importance as at the moment no genetic tools are currently available to transform ER. Our data open a window of opportunity for further *in vivo* or *in vitro* studies to investigate the role of specific proteins. By using bidimensional electrophoresis, we also highlight the importance of protein proteoforms (and PTMs) in ER biology, namely in virulence. We are currently performing PTM mapping using several ER strains with different levels of virulence. As these results and outcomes constitute a post-genome reference study on ER, we believe they could be of general relevance for the biology and the mechanistic basis of pathogenesis and attenuation phenomena in other *Rickettsiales* with high impact in human and animal health.

## Supporting Information

S1 Fig Representative growth kinetics of ERGvir and ERGatt obtained by (A) real time PCR targeting map-1 gene (dashed arrows represent the time of total medium exchange) and (B) reverse phase microscopy (N stands for nuclei, M for morula and EBs for elementary bodies). (PDF)

S2 Fig Daily clinical scores of the animals infected with ERGatt (0541 and 0614) and ERGvir (0212) strains. (PDF)

S3 Fig 1DE-SDS-PAGE protein migration profiles of the four biological replicates used for ERGvir and ERGatt strains within this work. The molecular marker profile is presented in the first lane.
(PDF)

S4 Fig Conventional 2D electrophoretic map of ERGatt proteins expressed at 96hpi in BAE cells. The crude extract of EBs were separated using a non-linear pH 3–10 IPG strip in the first dimension, followed by a pre-cast 12% SDS–PAGE in the second dimension. A representative gel of 3 biological replicates assays is shown.
(PDF)

S5 Fig Spots differentially expressed between ERGvir and ERGatt strains according to DIGE experiments (using 4 biological replicates per strain) and after image analysis using Samespots software analysis (A) and PCA results (B). (PDF)

S1 File Protein identification by MALDI-TOF/TOF. (PDF)

S1 Table Proteins common to both strains (core proteome) detected by 1DE-nanoLC-MALDI-TOF/TOF. Three biological replicates per strain were independently used and the results analyzed with Peaks software (using using simultaneously Peaks DB, MASCOT and X!Tandem algorithms). For each protein, columns denote the accession number, species, corresponding accession number in ERGardel strain, protein name, ordered locus name, gene name, function, Peaks software protein score (%), sequence coverage (%), number of peptides matched per identified protein, and the number of unique peptides.
(XLSX)

S2 Table Strain-specific proteins identified in ERGvir strain by 1DE-nanoLC-MALDI-TOF/TOF. Three biological replicates per strain were independently used and the results analyzed with Peaks software (using using simultaneously Peaks DB, MASCOT and X!Tandem algorithms). For each protein, columns denote the accession number, species, corresponding accession number in ERGardel strain, protein name, ordered locus name, gene name, function, Peaks software protein score (%), sequence coverage (%), number of peptides matched per identified protein, and the number of unique peptides.
(XLSX)

S3 Table List of strain-specific proteins detected in ERGatt strain by 1DE-nanoLC-MALDI-TOF/TOF. Three biological replicates per strain were independently used and the results analyzed with Peaks software (using using simultaneously Peaks DB, MASCOT and X!Tandem algorithms). For each protein, columns denote the accession number, species, corresponding accession number in ERGardel strain, protein name, ordered locus name, gene name, function, Peaks software protein score (%), sequence coverage (%), number of peptides matched per identified protein, and the number of unique peptides.
(XLSX)

S4 Table ERG proteins identified by conventional 2DE-MALDI-TOF/TOF (data compiled using MDM software). Spot numbers correspond to those indicated in S3 Fig. For each protein spot, columns denote the accession number in ERGardel strain, protein name, ordered locus name, gene name, function, theoretical isoelectric point (pI) and molecular masses (MM), protein scores, sequence coverage (%) and the number of peptides matched.
(XLSX)

S5 Table Proteins differentially 15344040expressed between ERGvir (A) and ERGatt (B) strains according to DIGE experiments. Spot numbers correspond to those indicated in S4 Fig. For each protein spot, columns denote the accession number in ERGardel, protein name, ordered locus name, gene name, function, protein scores, sequence coverage (%), the number of peptides matched, the average normalized volume for ERGvir and ERGatt replicates, the p-value and the fold change.
(XLSX)

## Abbreviations

ER: *Ehrlichia ruminantium*
ERGvir: *Ehrlichia ruminantium* Gardel virulent strain
ERGatt: *Ehrlichia ruminantium* Gardel attenuated strain
1DE: standard SDS-PAGE
nanoLC: nano-scale liquid chromatography
2DE: Bidimensional electrophoresis
PTM: post-translational modification
SPG: sucrose-phosphate-glutamate buffer
DIGE: Differential In-Gel Electrophoresis
