# Supplementary material for: Comparative Proteomic Profiling of Ehrlichia ruminantium Pathogenic Strain and Its High-Passaged Attenuated Strain Reveals Virulence and Attenuation-Associated Proteins
Source: PLoS One. 2015 Dec 21;10(12):e0145328. doi: 10.1371/journal.pone.0145328 (PMC4686967; doi:10.1371/journal.pone.0145328)

S1 Fig . Representative growth kinetics of ERGvir and ERGatt obtained by (A) real time PCR targeting map-1 gene (dashed arrows represent the time of total medium exchange) and (B) reverse phase microscopy (N stands for nuclei, M for morula and EBs for elementary bodies).

(A)

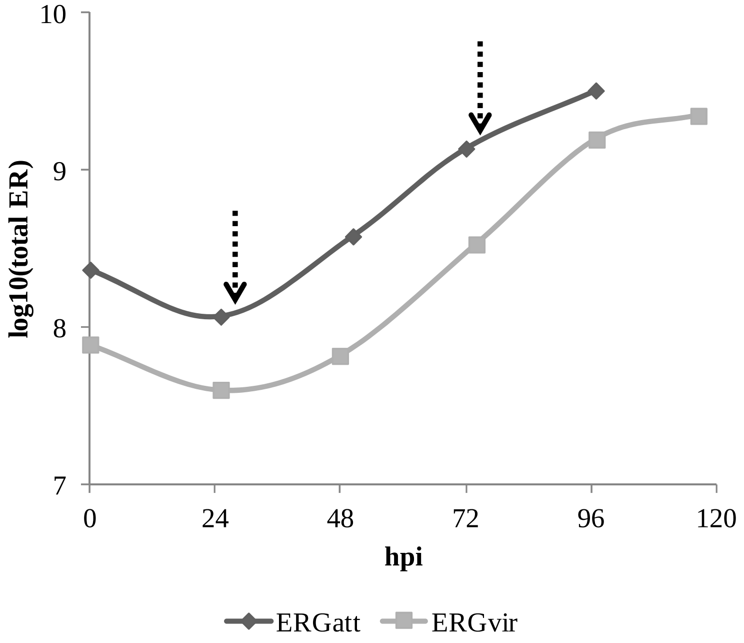

(B)

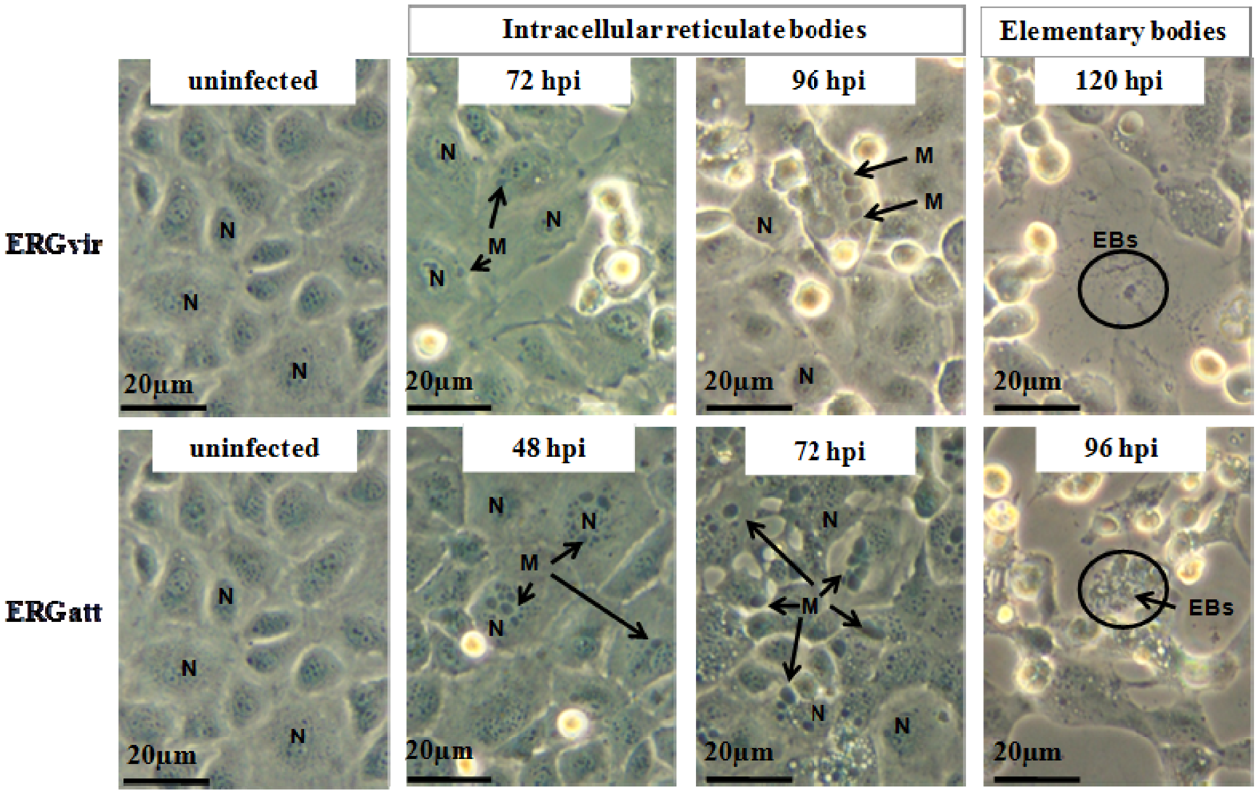

Supplement: S1 Fig — (PDF) [file pone.0145328.s001.pdf]
