# Supplementary figures and images for: Comparative Proteomic Profiling of Ehrlichia ruminantium Pathogenic Strain and Its High-Passaged Attenuated Strain Reveals Virulence and Attenuation-Associated Proteins
Source: PLoS One. 2015 Dec 21;10(12):e0145328. doi: 10.1371/journal.pone.0145328 (PMC4686967; doi:10.1371/journal.pone.0145328)

**S2 Fig.** Daily clinical scores of the animals infected with ERGatt (0541 and 0614) and ERGvir (0212) strains.

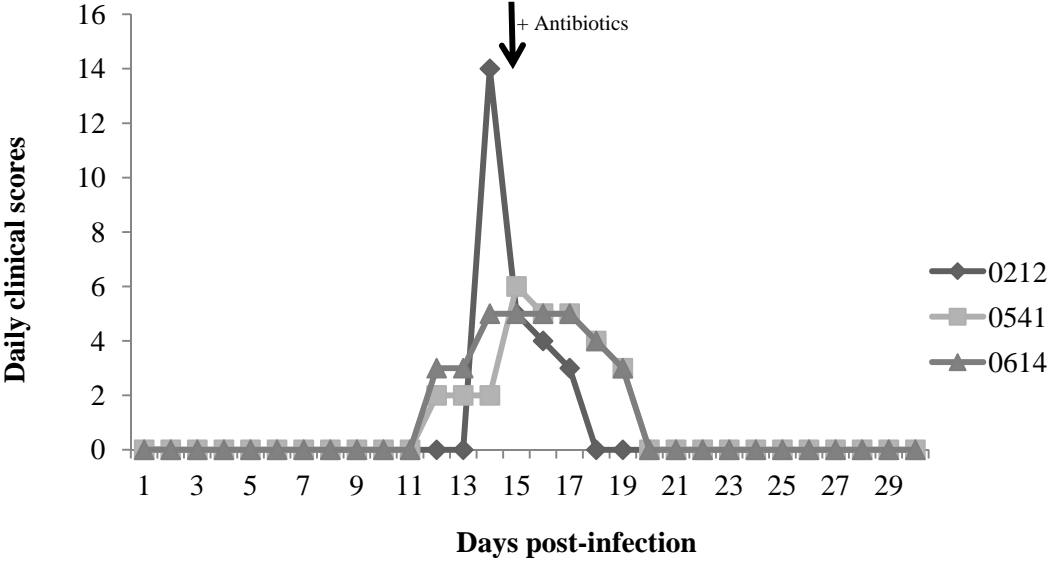

Supplement: S2 Fig — (PDF) [file pone.0145328.s002.pdf]
