# Supplementary material for: Comparative Proteomic Profiling of Ehrlichia ruminantium Pathogenic Strain and Its High-Passaged Attenuated Strain Reveals Virulence and Attenuation-Associated Proteins
Source: PLoS One. 2015 Dec 21;10(12):e0145328. doi: 10.1371/journal.pone.0145328 (PMC4686967; doi:10.1371/journal.pone.0145328)

S3 Fig. 1DE-SDS-PAGE protein migration profiles of the four biological replicates used for ERGvir and ERGatt strains within this work. The molecular marker profile is presented in the first lane.

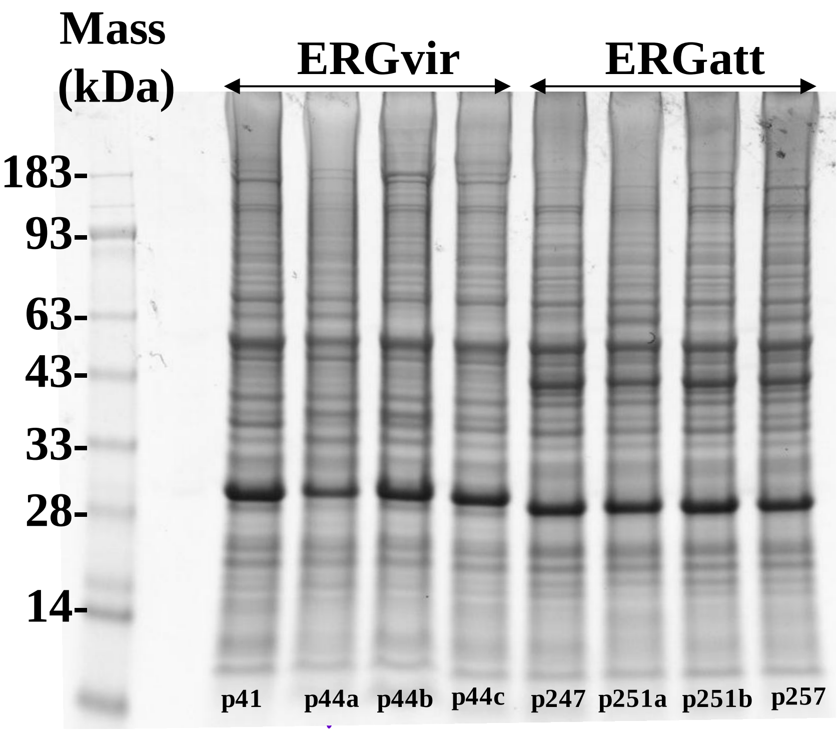

Supplement: S3 Fig — The molecular marker profile is presented in the first lane. (PDF) [file pone.0145328.s003.pdf]
