# Supplementary material for: Comparative Proteomic Profiling of Ehrlichia ruminantium Pathogenic Strain and Its High-Passaged Attenuated Strain Reveals Virulence and Attenuation-Associated Proteins
Source: PLoS One. 2015 Dec 21;10(12):e0145328. doi: 10.1371/journal.pone.0145328 (PMC4686967; doi:10.1371/journal.pone.0145328)

S4 Fig. Conventional 2D electrophoretic map of ERGatt proteins expressed at 96hpi in BAE cells. The crude extract of EBs were separated using a non-linear pH 3–10 IPG strip in the first dimension, followed by a pre-cast 12% SDS–PAGE in the second dimension. A representative gel of 3 biological replicates assays is shown.

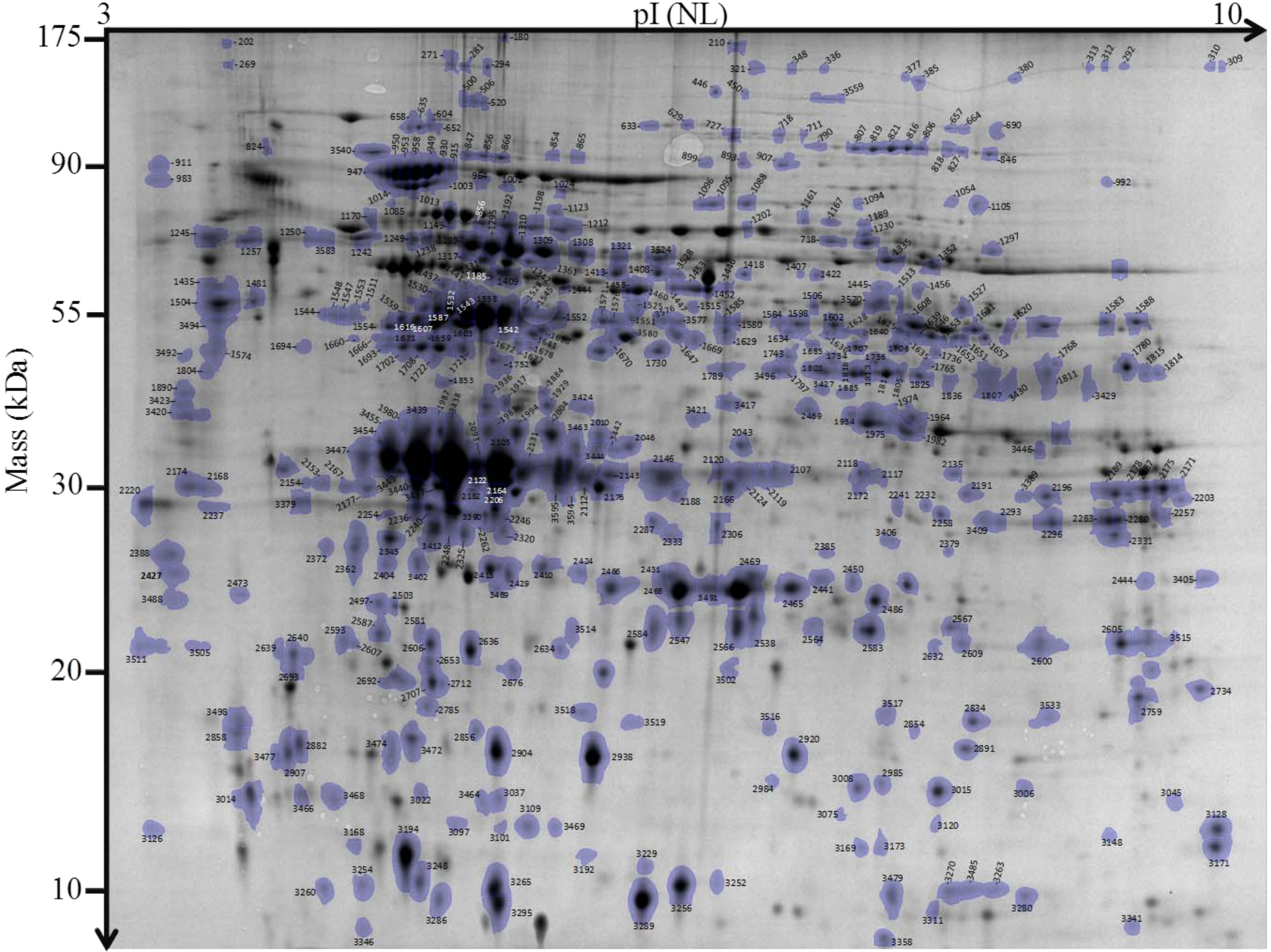

Supplement: S4 Fig — The crude extract of EBs were separated using a non-linear pH 3–10 IPG strip in the first dimension, followed by a pre-cast 12% SDS–PAGE in the second dimension. A representative gel of 3 biological replicates assays is shown. (PDF) [file pone.0145328.s004.pdf]
