# Supplementary material for: Comparative Proteomic Profiling of Ehrlichia ruminantium Pathogenic Strain and Its High-Passaged Attenuated Strain Reveals Virulence and Attenuation-Associated Proteins
Source: PLoS One. 2015 Dec 21;10(12):e0145328. doi: 10.1371/journal.pone.0145328 (PMC4686967; doi:10.1371/journal.pone.0145328)

S5 Fig. Spots differentially expressed between ERGvir and ERGatt strains according to DIGE experiments (using 4 biological replicates per strain) and after image analysis using Samespots software analysis (A) and PCA results (B).

(A)

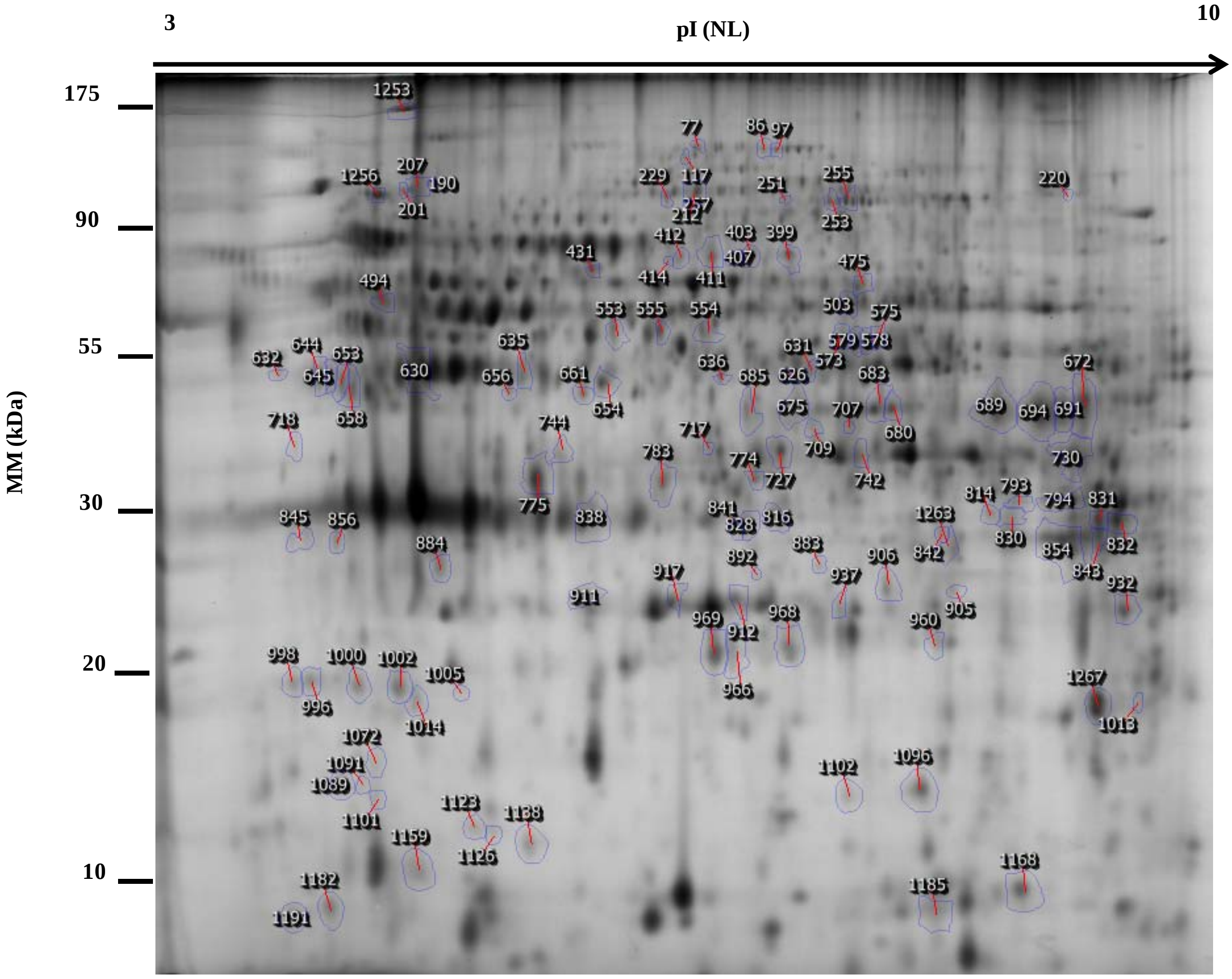

(B)

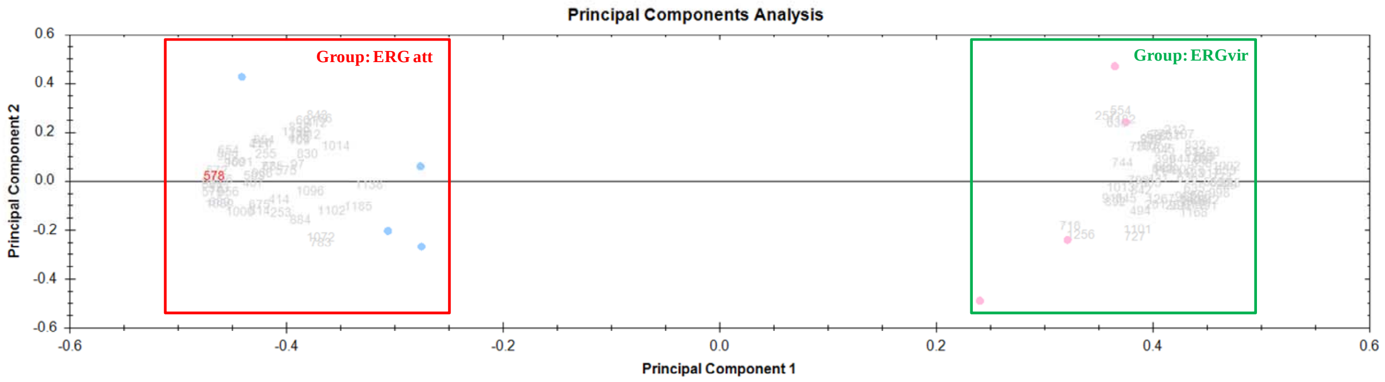

Supplement: S5 Fig — (PDF) [file pone.0145328.s005.pdf]
