# Supplementary material for: Comparative Proteomic Profiling of Ehrlichia ruminantium Pathogenic Strain and Its High-Passaged Attenuated Strain Reveals Virulence and Attenuation-Associated Proteins
Source: PLoS One. 2015 Dec 21;10(12):e0145328. doi: 10.1371/journal.pone.0145328 (PMC4686967; doi:10.1371/journal.pone.0145328)
Supplement: S1 File — (PDF) [file pone.0145328.s006.pdf]

S1 file - Protein identification by MALDI-TOF/TOF

The MS/MS spectra obtained for the samples processed by 1DE-nanoLC were searched against database search using PEAKS search engine tool (PEAKS Studio 5.3; Bioinformatics Solutions Inc., Waterloo, ON, Canada) [33], combining 3 search engines MASCOT, X!Tandem and Peaks DB. The search parameters for the MS/MS spectra are presented below:

- MASCOT search:

**Database**	*Ehrlichia ruminantium* with proteoforms was used (December 2014, total 2386 entries)
**Taxonomy**	Bacteria (eubacteria)
**Enzyme**	Trypsin;
**Maximum missed cleavage**	2
**Variable modifications**	Carbamidomethyl, deamidated and oxidation
**Error tolerance**	0
**Parent mass error tolerance**	50ppm
**Fragment mass error tolerance**	0.5Da
**Charge**	+1
**Precursor mass search type**	monoisotopic
**Valid protein candidates**	When global Mascot score was greater than 60 with a significance level of $p<0.05$.

Note: The same parameters were used for protein identification in spots excised from 2D gels

- XTandem:

**Reverse database sequence search**	false
**fragment monosioptic mass error tolerance**	0.5Da
**Contrast angle used**	no
**Degree of contrast angles**	40
**Residue modification mass**	57.021464 for Cys; defined potential modification: 15.994915 for Met and 0.984016 for Asn
**Expected value**	1
**Point mutations**	No

- Peaks DB version 5.3

**Parent mass error tolerance**	50ppm
**Fragment mass error tolerance**	0.5Da
**Precursor mass search type**	monoisotopic
**Enzyme**	Trypsin;
**Maximum missed cleavage**	2
**Modifications**	Oxidation M: 15.99; deamidation: 0.98; carbamidomethylation: 57.02;
**Max variable PTM per peptide mass**	3
**Database**	E.rum
**Taxonomy**	All
**Searched entry**	2386
**De novo dependencies**	199
**Decoy performed**	true
